# SWI/SNF complexes modulate gene expression and the development of physical dependence to ethanol

**DOI:** 10.1111/acer.70223

**Published:** 2026-01-12

**Authors:** Laura D. Mathies, GinaMari G. Blackwell, Andrew G. Davies, Jill C. Bettinger

**Affiliations:** ^1^ Department of Pharmacology and Toxicology Virginia Commonwealth University Richmond Virginia USA; ^2^ Virginia Commonwealth University Alcohol Research Center Richmond Virginia USA

**Keywords:** alcohol dependence, alcohol withdrawal, *Caenorhabditis elegans*, differential gene expression, SWI/SNF chromatin remodeling complex

## Abstract

**Background:**

Long exposure to ethanol causes the development of physical dependence, which causes withdrawal symptoms when ethanol is removed. We exploited the rapid development of physical dependence in *Caenorhabditis elegans* (*C. elegans*) to examine the ethanol‐induced transcriptional changes that correspond with physical dependence and withdrawal effects.

**Methods:**

After an 18‐h exposure to an intoxicating concentration of ethanol, we observe the development of physical dependence; withdrawal from ethanol causes an increase in their preference for thicker parts of a bacterial lawn, a behavior we term withdrawal‐induced bordering (WIB). We performed transcriptional analysis to identify genes whose expression changes correlate with WIB.

**Results:**

We found that WIB is transient, resolving within 6 h of removal from ethanol, suggesting it is driven by short‐term gene expression changes. We identified 1870 genes with differential expression immediately after ethanol exposure but not after 6 h of withdrawal; these are candidate mediators of WIB. We found that the SWI/SNF chromatin remodeling complex, known to be important in the response to acute ethanol exposure, is required for normal WIB. Loss of *swsn‐9* attenuated but did not eliminate WIB, suggesting that there are *swsn‐9*‐dependent and *swsn‐9*‐independent components of WIB. Regulation of 1031 ethanol‐responsive genes requires *swsn‐9*. WIB phenocopies reduced *npr‐1* activity, and two genes implicated in the *npr‐1* signaling pathway, *jmjc‐1* and *dod‐24*, were transiently regulated after extended ethanol exposure. *dod‐24* mutants have attenuated WIB.

**Conclusions:**

Extended exposure to ethanol causes the development of transient physical dependence in *C. elegans*, and the SWI/SNF complex is involved in this process. Genes involved in fatty acid metabolism were regulated over the WIB timecourse, and *dod‐24*, which is involved in temperature sensitivity, is required for normal WIB. Together, these results suggest a model in which modulation of lipid membrane composition may be one mechanism for the development of physical dependence on ethanol.

## Graphical abstract

The development of physical dependence on ethanol is an important component of alcohol addiction because dependent individuals experience withdrawal symptoms during acute abstinence. This study used *C. elegans* to identify gene expression changes accompanying physical dependence. SWI/SNF chromatin remodeling contributed significantly to dependence. Regulation of lipid genes, including some involved in temperature responses, was correlated with withdrawal. This suggests that these responses may share molecular mechanisms and may point to a role for modifying membrane structure in developing physical dependence.

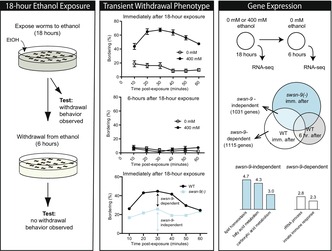

## INTRODUCTION

Alcohol is one of the most widely used psychoactive substances in the world. In 2022, approximately 50% of the US population aged 12 or older reported using alcohol in the last month and 5.7% were heavy alcohol users (Substance Abuse and Mental Health Services Administration, 2023). Chronic alcohol use leads to changes in neuronal function that produce physical dependence; dependence is revealed by withdrawal symptoms upon cessation of alcohol use. Avoidance of withdrawal is important in continued pathological alcohol use and contributes to alcohol use disorder (AUD) (Koob et al., 2020).

Long‐term changes in gene expression in response to drug exposure are one mechanism by which physical dependence develops. Experience‐dependent gene expression modulation can be achieved through epigenetic regulation; this is thought to be an important factor in humans in the development of alcohol dependence and AUD (Hillemacher, 2011; Krishnan et al., 2014; Marballi et al., 2014; Pandey et al., 2017; Starkman et al., 2012). Epigenetic regulators alter gene expression by manipulating covalent modifications of histone proteins, changing the methylation state of DNA, or by moving nucleosomes to remodel the chromatin structure. These gene expression changes can be maintained over long periods of time, providing a molecular memory of alcohol consumption and contributing to the etiology of the disease. Epigenetic regulation has been implicated in alcohol‐dependent gene expression changes (Barbier et al., 2015; Barker et al., 2013; Moonat et al., 2013; Pandey et al., 2008; Sakharkar et al., 2014; You et al., 2014), the development of rapid tolerance to ethanol (Ghezzi et al., 2013; Sakharkar et al., 2012; Wang et al., 2007), and drinking behaviors in animal models (Barbier et al., 2015; Sakharkar et al., 2014; Warnault et al., 2013; Wolstenholme et al., 2011). Here, we investigate the role of SWI/SNF chromatin remodeling in the regulation of gene expression and the physiological effects of prolonged ethanol exposure.

SWI/SNF chromatin remodelers are multi‐protein complexes that use the energy generated by ATP hydrolysis to alter the locations of nucleosomes, thereby revealing or occluding regulatory sequences and changing gene expression. The complexes contain core and accessory subunits that combine to produce molecularly and functionally distinct complexes. Two major classes of SWI/SNF are BAF (Brm/Brg1‐associated factors) and PBAF (Polybromo‐associated BAF) (Kwon & Wagner, 2007; Mohrmann et al., 2004; Mohrmann & Verrijzer, 2005). We have previously examined SWI/SNF complexes for effects on the acute level of response, since this is a predictor of AUD (Mathies et al., 2015). We found that SWI/SNF complex genes are important for determining the physiological level of response to ethanol in worms and variation in SWI/SNF genes is associated with alcohol dependence and other alcohol‐related phenotypes in humans (Mathies et al., 2015, 2017).

Upon extended exposure to ethanol, *C. elegans* develops physical dependence that is revealed when the ethanol is withdrawn. Acute ethanol exposure potentiates the function of the NPY‐like *npr‐1* signaling pathway, and when adult animals are exposed to ethanol for 18 h, they downregulate the function of this pathway. Here, we use the term *withdrawal* to describe the process of removing the animals from ethanol after this extended (18‐h) exposure. We define *physical dependence* as the functional changes made by animals in response to prolonged exposure to ethanol; these changes are revealed when the drug is removed as new, withdrawal‐induced phenotypes, indicating that the animals require the continued presence of the drug for normal function. Prolonged ethanol exposure causes the downregulation of the *npr‐1* signaling pathway. This downregulation becomes evident when the worms are withdrawn from ethanol, as they show an increased preference for thicker parts of the bacterial lawn (bordering behavior) in an *npr‐1*‐dependent manner (Davies et al., 2004). We refer to this as withdrawal‐induced bordering (WIB). Animals that are withdrawn from 20 h of ethanol exposure also demonstrate slower locomotion speed than untreated controls, which can be rescued by re‐exposure to ethanol; this exposure paradigm results in downregulation of the expression of a SLO*‐*1 BK channel translational reporter and withdrawal‐induced slowing could be decreased by overexpression of the SLO*‐*1 BK channel (Scott et al., 2017). Together, these observations suggest that extended ethanol exposure in worms induces gene expression changes that underlie the development of physical dependence on ethanol.

We use withdrawal‐induced bordering behavior to probe the molecular mechanisms underlying the development of physiological tolerance to ethanol. Here, we examine behavior and gene expression following an extended ethanol exposure and during ethanol withdrawal in *C. elegans*. We find that the effects of ethanol withdrawal on bordering behavior are transient; withdrawal‐induced behavioral changes are reversed by 6 h after removal from ethanol. We therefore examine gene expression immediately after extended ethanol exposure and again at 6 h of withdrawal when behavior has normalized. We identify 1870 genes whose expression is altered immediately after ethanol exposure, but is normalized following a 6‐h recovery period. We predicted that at least some of these changes in gene expression may be regulated by the SWI/SNF chromatin remodeling complex, and we show that the SWI/SNF gene *swsn‐9* is required for the degree of WIB. This indicates that there are *swsn‐9*‐dependent and *swsn‐9*‐independent components of the response to extended ethanol exposure. We identify 1031 genes whose regulation following ethanol exposure requires *swsn‐9*. Two of the transiently regulated genes have been implicated in neuropeptide Y‐like signaling; we find that one of the genes, *dod‐24*, is required for withdrawal‐induced behavioral changes.

## MATERIALS AND METHODS

### Strains

*Caenorhabditis elegans* strains were cultured as described previously (Brenner, 1974; Wood, 1988). All strains were grown at 20°C unless otherwise specified and were derived from the Bristol strain N2. Strains were obtained from the *Caenorhabditis* Genetics Center or the National BioResource Project. The following alleles were used in this study: *LGIII: swsn‐9(ok1354)* (Large & Mathies, 2014), *jmjc‐1(tm3525)* (Kirienko & Fay, 2010); *LGIV: dod‐24(ok2629)* (*C. elegans* Deletion Mutant Consortium, 2012). *LGX: npr‐1(ky13)* (de Bono & Bargmann, 1998). All strains were outcrossed to N2 six times before behavioral analysis. Deletions were tracked using PCR with primers flanking the deleted region. Homozygosity was confirmed using PCR with one primer internal to the deletion. Primers and strains are listed in File S1. Double mutants were generated using standard genetic crossing techniques.

### Withdrawal‐induced bordering (WIB)

#### Ethanol exposure

NGM plates were seeded with OP50 bacteria on one half of the plate, cultured at room temperature overnight, and dried for 2 h at 37°C. Plates were weighed, and ice‐cold 100% ethanol was added to the unseeded side of the plate to generate a final concentration of 0 mM or 400 mM ethanol, and the plates were kept at a slight angle to prevent the ethanol from contacting the bacteria directly. The plates were sealed with Parafilm, and the ethanol was allowed to absorb into the plate for 30 min. Approximately 100 L4‐stage worms from healthy, unstarved populations were placed on ethanol‐treated or control plates; the plates were sealed with Parafilm and incubated at 20°C for 18 h. During this time, the animals mature into young adults; all behavioral assays were performed on first‐day adult animals. For samples that were removed from ethanol prior to testing, the worms were transferred to plates seeded with OP50 and incubated at 20°C for 6 h prior to behavioral or gene expression analysis.

#### Bordering assays

Assay plates were seeded to the edge of the plate with OP50 and incubated at room temperature overnight to obtain a thin bacterial lawn. Copper rings were embedded in the plates and an approximately 1 mm clump of thick OP50 was placed in the center of each ring to produce an area of thick bacteria on an otherwise thin bacterial lawn; this mimics the thicker border of an OP50 seeded plate. Control and ethanol‐treated worms were assayed in different rings on the same plate. Twenty worms were placed in each ring away from the thick bacterial spot. Immediately after placing the worms in the rings, time‐lapse movies were recorded at 1 frame/s for 1 h using Image Pro Plus version 10.0.12 (Media Cybernetics, Inc., Rockville, MD). Bordering was assessed in the movies at 10‐min intervals by a researcher blinded to the treatment condition. The number of worms outside of the bacterial spot (*n*) was used to infer the number of worms in the spot (20—*n*); worms with >50% of their body in contact with the bacterial spot were considered to be in the spot. All statistical comparisons were made in Prism version 9.5.1 (GraphPad Software, San Diego, CA). To compare treatment (400 mM ethanol) to control (0 mM ethanol) within a genotype across the time course, we used two‐way ANOVA or mixed‐effects analysis, depending on whether or not there was missing data. Withdrawal‐induced bordering (WIB) is an increase in bordering after withdrawal from ethanol exposure. To compare the degree of WIB between strains, we calculated the area under the curve and used one‐way ANOVA to compare genotypes and treatments. Post hoc tests were performed with Holm‐Šídák multiple comparisons *p*‐value correction.

#### Rescuing WIB with re‐exposure to ethanol

Approximately 80 wild‐type worms were exposed to 0 or 400 mM ethanol for 18 h, as described above. Immediately following the exposure, 20 worms from each treatment population (0 mM and 400 mM; Group 1) were assessed for bordering behavior every 10 min using a dissecting microscope. At the same time, an additional 40 worms from each treatment population (Group 2) were transferred to plates containing a thin lawn of bacteria and 0 mM ethanol, thus beginning ethanol withdrawal. After 20–25 min of withdrawal, when the original population (Group 1) of 400 mM‐treated worms exhibited WIB, 20 worms from each treatment population in Group 2 were moved to assay plates containing 0 mM ethanol (continued withdrawal) and 20 were moved to 400 mM ethanol (re‐exposure to ethanol). Two‐minute videos were recorded at 1 frame per second, spanning the 10‐, 20‐, and 30‐min timepoints. Bordering assay plates were prepared and bordering was assessed as described above.

#### Suppression of bordering in *npr‐1* mutants

Bordering assay plates were prepared as described above. Twenty worms were assessed for bordering behavior every 10 min using a dissecting microscope. Double mutants were assayed in parallel with N2 wild type and each single mutant. Statistical comparisons were made at the 30‐min timepoint using paired Student's *t*‐tests.

### Internal ethanol assays

This assay was performed as described previously (Mathies et al., 2015). Worms were exposed to 400 mM ethanol for 18 h. One hundred worms were picked directly from the exposure plates into 10 μL of 25% TCA on ice. An additional 100 worms were moved to plates containing 0 mM ethanol for 30 min and then picked into 10 μL of 25% TCA on ice. One microliter of sample was added to 200 μL of alcohol reagent (Pointe Scientific) in a flat bottom 96‐well culture plate on ice. The reactions were incubated at 37°C for 5 min prior to reading absorbance at 340 nm using an Epoch microplate reader with the Gen6 v. 1.03 software (BioTek). A standard curve was prepared in 25% TCA to account for any effect that TCA might have on the assay reagent. All measurements were performed in triplicate, and the average was used for calculations. Three independent samples were assayed, and the mean (±SEM) was calculated for each genotype and time point. Worm volumes were estimated from images taken using ImagePro version 10.0.12 on a Leica MZ6 microscope with a 1× objective and 4× magnification. Images were analyzed using ImageJ version 1.53 t with the SmartRoot plugin (Lobet et al., 2011). Images of at least 10 worms were used to calculate an average worm volume. Statistical comparisons were made using two‐way ANOVA.

### RNA sequencing

#### Sample collection

RNA was prepared from biological replicates (*n* = 5) that were collected on different days; control and ethanol exposures were always done in parallel. Approximately 100 worms were exposed to 0 mM or 400 mM ethanol as described for withdrawal‐induced bordering. Immediately following treatment, the worms were washed from the plates with M9 medium, rinsed once with M9, and stored in Trizol (Ambion, Carlsbad, CA) at −80°C until RNA preparation. RNA was isolated using the miRNeasy kit with DNase I digestion performed on the column (Qiagen, Venlo, Netherlands). RNA integrity was assessed on representative samples using the Bioanalyzer 2100 with the High Sensitivity RNA kit (Agilent, Santa Clara, CA). All samples had RIN scores greater than 9.5.

#### RNA sequencing and analysis

The RNA was polyA selected, and indexed sequencing libraries were prepared and sequenced by GeneWiz (South Plainfield, NJ). The libraries were sequenced as 150‐base, paired‐end reads to an average read depth of 17 million reads per sample using the Illumina HiSeq platform (Illumina, San Diego, CA). We performed initial quality control using FastQC (https://www.bioinformatics.babraham.ac.uk/projects/fastqc/) and found that the sequencing libraries had high quality scores and did not contain overrepresented sequences indicative of Illumina adapters. The sequences were aligned to the *C. elegans* genome (Ensembl genome assembly release WBcel325) using STAR version 2.6.0a (Dobin et al., 2013) with the following parameters specified (quantMode GeneCounts, outFilterMultimapNmax 1, alignIntronMin 40, alignIntronMax 18,000, outFilterMismatchNoverReadLmax 0.02). Aligned reads were sorted and indexed using SAMtools (Li et al., 2009). The STAR aligner generated gene‐based read counts, which we used for differential expression analysis in DESeq2 version 1.18.1 (alpha = 0.05) (Love et al., 2014). For wild‐type gene expression, all N2 samples were analyzed together. For *swsn‐9* gene expression, all N2 samples were re‐analyzed with the *swsn‐9* samples. Contrasts were used to identify differentially expressed genes (DEGs) for a particular sample type. A comparison of N2 DEGs from the wild‐type and *swsn‐9* analyses is included in File S5.

To examine the variance among our samples and replicates, we performed principal component analysis using the rlogTransformation and plotPCA functions in DESeq2. Volcano plots and heatmaps were generated in R version 3.6.3 using RStudio version 1.4.1717 (RStudio, Boston, MA). Heatmaps were produced with the pheatmap package using variance‐stabilized counts from DESeq2. Counts were centered and scaled by gene (*z*‐scores). Only samples from the contrasted groups were included. WormBase IDs were mapped to gene symbols using the org.Ce.eg.db annotation package. Gene‐specific statistical power was estimated using the RNASeqPower package. Mean normalized counts and gene‐wise dispersions were obtained from DESeq2, and median library depth across the samples in each group was calculated. When the per‐gene dispersion was not available due to very low expression, the fitted mean dispersion trend from DESeq2 was used. Power was calculated for detecting a 1.5‐fold change between the treated and untreated groups at a significance level of 0.05. Gene Ontology (GO) term analysis was performed using the statistical overrepresentation test in PANTHER (Mi et al., 2013, 2017; Thomas et al., 2003). These are found in File S3 (wild‐type N2) and File S6 (*swsn‐9*). Gene lists were analyzed using the GO biological process complete annotation with Fisher's exact test with false discovery rate (FDR) correction. The background list included all genes with an average read count of one across all samples being analyzed.

## RESULTS

We have previously observed that prolonged ethanol exposure alters bordering behavior, which is the tendency for animals to spend time in the thicker parts of the bacterial lawn. This behavior is driven by a preference for lower oxygen concentration (Gray et al., 2004). The laboratory wild‐type strain N2 exhibits low levels of bordering under normal culture conditions. However, N2 worms that have been exposed to ethanol for 18–22 h, and then withdrawn from ethanol, significantly increase their bordering behavior; this is dependent on the *npr‐1* pathway (Davies et al., 2004). We refer to this change in behavior as withdrawal‐induced bordering (WIB). We use WIB as a model for identifying the gene expression changes and physiological effects of prolonged ethanol exposure.

**FIGURE 1:**
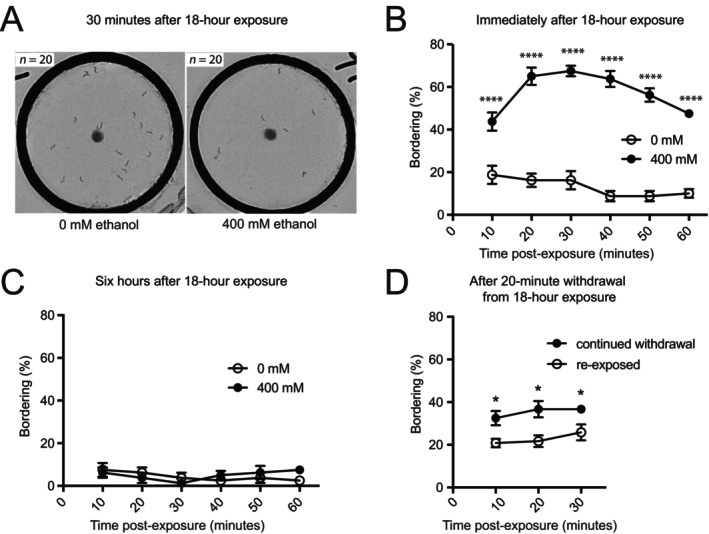
Following prolonged ethanol exposure, worms exhibit a transient withdrawal phenotype, withdrawal‐induced bordering (WIB). (A) Wild‐type N2 worms (*n* = 20) treated with either 0 mM (left image) or 400 mM (right image) ethanol for 18–20 h were placed within copper rings on plates that were seeded thinly with bacteria and contained no ethanol. A thicker clump of bacteria was placed in the center of the ring, mimicking the dense border of a bacterial lawn. Worms in the bacterial clump are less visible, so bordering behavior is scored by counting the number of worms outside of the clump and subtracting from 20. Images were taken ~30 min postexposure; the full video is available as Video S1. Worms that had been treated with 0 mM ethanol tended to remain outside of the bacterial clump, while worms that had been treated with 400 mM ethanol were more frequently found in the bacterial clump. (B, C) Bordering assays performed on wild*‐*type N2 worms. Filled symbols indicate prior 18‐h exposure to 400 mM ethanol; open symbols indicate prior exposure to 0 mM ethanol. All genotypes and/or conditions on the same graph were tested simultaneously on the same plates. (B) Wild*‐*type N2 worms exhibited an increase in bordering behavior between 10 and 60 min following withdrawal from an 18‐h exposure to 400 mM exogenous ethanol (*n* = 4). Effect of treatment: *P* ≤ 0.0001. (C) Wild*‐*type N2 worms exposed to 400 mM ethanol for 18 h and then withdrawn from ethanol for 6 h prior to the assay did not show an increase in bordering behavior (*n* = 4). (D) Wild‐type N2 worms were exposed to 400 mM ethanol for 18 h, moved to ethanol‐free plates for 20–25 min, then moved to plates with 0 mM (continued withdrawal) or 400 mM ethanol (re‐exposed), and tested for bordering behavior. The multiple manipulations resulted in an overall decrease in bordering behavior. Re‐exposure to ethanol nonetheless suppressed bordering behavior. Effect of assay condition: *P* = 0.0023. Statistical comparisons were made using two‐way ANOVA with Holm‐Šídák post hoc tests. ****p ≤ 0.0001, *p ≤ 0.05. Error bars indicate SEM.

We measured the time course of WIB using our previously established assay (Davies et al., 2004). We exposed N2 worms to 0 or 400 mM ethanol for 18 h on plates that contained bacteria for feeding. The worms were then transferred to plates containing a thin lawn of bacteria with a thicker spot of bacteria in the center of a copper ring. The copper ring serves as a corral that allows us to test multiple genotypes or treatments on the same plate. The bacterial spot mimics the thicker border of a bacterial lawn and is used to quantify bordering behavior. Worms treated with 0 and 400 mM ethanol were always assayed in parallel on the same plates. To determine the time course of the behavior, we recorded videos continuously for 1 h following removal from 0 or 400 mM ethanol and we assessed the number of bordering worms every 10 min. N2 worms that were not exposed to ethanol spent most of their time outside of the bacterial spot, whereas N2 worms that were withdrawn from 400 mM ethanol spent significantly more time in the bacterial spot (Figure 1A). The percentage of bordering worms was significantly different between worms exposed to 400 and 0 mM ethanol at times ranging from 10 to 60 min postexposure (Figure 1B); this is WIB. We observed the greatest WIB 30 min after removal from ethanol, consistent with our previous work (Davies et al., 2004). Next, we asked how long this withdrawal phenotype persisted by removing the worms from ethanol for 6 h prior to performing the assay. After this extended withdrawal period, we did not observe an increase in bordering in the ethanol‐treated worms (Figure 1C), indicating that WIB is an acute and transient withdrawal phenotype.

One characteristic of drug withdrawal behavior is that it can be suppressed with re‐exposure to the drug. We tested whether our bordering behavior could be suppressed by re‐exposing animals to ethanol after they had begun to demonstrate WIB (Figure 1D, Figure S1). We exposed animals to 0 and 400 mM ethanol for 18 h and then transferred a subset of the animals to bordering assay plates to observe WIB (Figure S1A). At the same time, another subset was transferred to ethanol‐free plates for 20–25 min to initiate withdrawal. We then moved them to bordering assay plates containing 0 mM (continued withdrawal) or 400 mM ethanol (re‐exposure). While the handling of the animals through the multiple steps of this experiment diminished overall bordering behavior, we found that re‐exposure to ethanol suppressed bordering behavior (Figure 1D). Furthermore, we detected WIB in animals that remained on 0 mM ethanol, but not in those re‐exposed to 400 mM ethanol (Figure S1B,C). These results support our conclusion that WIB is a withdrawal phenotype that reflects the development of physical dependence during prolonged ethanol exposure.

### Prolonged ethanol exposure causes significant changes in gene expression

We reasoned that WIB is likely to result, at least in part, from changes in gene expression that occur during prolonged ethanol exposure. We therefore sought to characterize the gene expression changes that occur following ethanol exposure with the goal of identifying genes that mediate this withdrawal phenotype.

**FIGURE 2:**
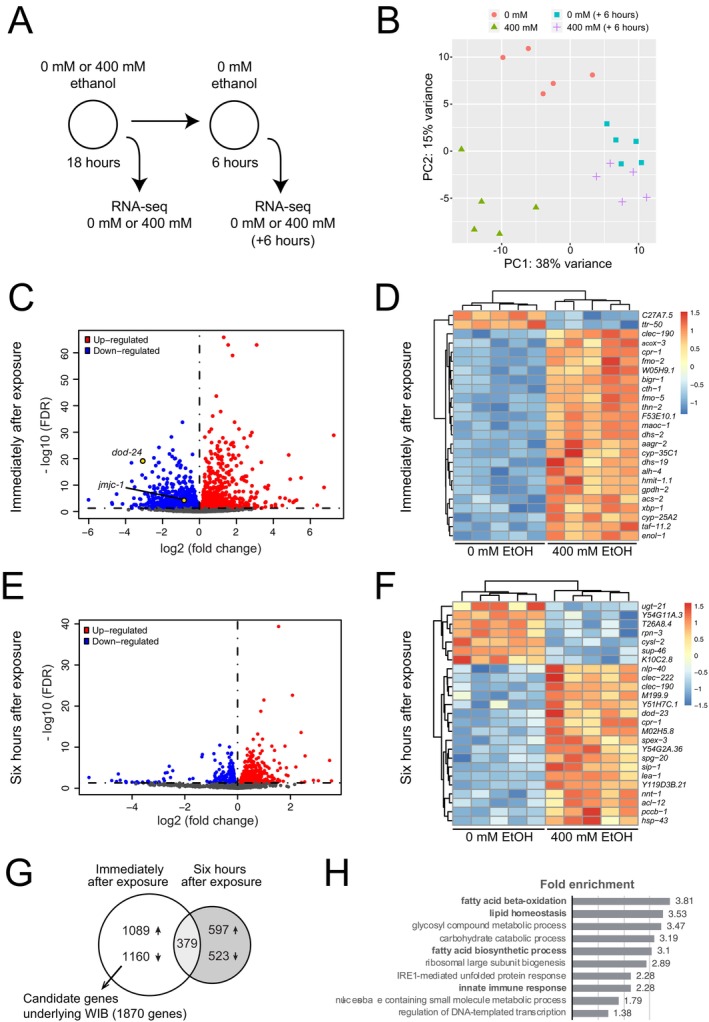
Prolonged ethanol exposure induces gene expression changes. (A) Wild‐type animals were exposed to either 0 mM or 400 mM ethanol for 18 h. RNA sequencing was performed immediately following exposure, or after a 6‐h withdrawal period. (B) Principal component analysis with gene expression based on the first two principal components (PC1 and PC2); replicates cluster together. The difference between samples treated with 0 and 400 mM ethanol is more pronounced immediately after ethanol exposure than it is 6 h later. (C–F) Gene expression results for wild‐type worms exposed to 400 mM ethanol versus 0 mM ethanol and harvested immediately after exposure (C, D) or 6 h later (E, F). (C, E) Volcano plots show upregulated (red) and downregulated (blue) genes; genes tested using mutations are highlighted in yellow. (D, F) Heat maps display the top 25 differentially expressed genes with blue indicating lower and red indicating higher expression. (G) Venn diagram showing the overlap between differentially expressed genes (DEGs) immediately following ethanol exposure and 6 h postexposure. Genes underlying WIB are predicted to be differentially expressed immediately after ethanol exposure but return to basal levels within 6 h; 1870 genes fit these criteria. (H) Overrepresented Gene Ontology (GO) biological process terms for DEGs identified immediately after exposure, but not 6 h postexposure.

We used RNA sequencing as an unbiased method to identify transcripts whose expression changed because of prolonged ethanol exposure. We exposed worms to either 0 or 400 mM ethanol for 18 h as described for the bordering assay. We anticipated that the ethanol‐induced gene expression changes underlying WIB would be normalized after a 6‐h withdrawal period off ethanol because we no longer see the behavior at this timepoint (Figure 1C). Therefore, we harvested worms either immediately following ethanol exposure or 6 h after removal from ethanol (Figure 2A). We performed five biological replicates on different days, and treatment and control exposures were done in parallel. RNA‐sequencing libraries were prepared and sequenced by GeneWiz (South Plainfield, NJ).

To examine the correlation among our samples and replicates, we performed principal component analysis. We found that our sample types grouped separately, with the difference between immediately postexposure and 6 h postexposure accounting for 38% of the variance (PC1) and ethanol treatment accounting for 15% of the variance (PC2) in the dataset (Figure 2B). The treatment and control samples were much more divergent when harvested immediately following exposure than they were 6 h postexposure, suggesting that many of the ethanol‐induced gene expression changes were reversed following removal from ethanol.

We compared gene expression in worms exposed to 0 or 400 mM ethanol under both exposure paradigms. When the worms were harvested immediately following ethanol exposure, we found 2249 genes that were differentially expressed between treatment and control (FDR < 0.05, fold change >1); approximately equal numbers of genes were upregulated as were downregulated (Figure 2C). The top 25 ethanol‐responsive genes are shown in the heatmap (Figure 2D); most of the genes are upregulated following ethanol exposure.

Six hours postexposure, we found 1120 genes that were differentially expressed between treatment and control (FDR < 0.05, fold change >1); approximately equal numbers of genes were upregulated as were downregulated (Figure 2E). The top 25 ethanol‐responsive genes at this timepoint are shown in the heatmap (Figure 2F); again, the majority of the genes are upregulated.

We compared the differentially expressed genes (DEGs) identified immediately off ethanol to those identified 6 h postexposure and found only 379 genes in common (Figure 2G), 291 of which were differentially expressed in the same direction. All DEG lists are in File S2. These stable gene expression changes may be important for some of the long‐term consequences of prolonged ethanol exposure, but they are unlikely to underlie WIB because this withdrawal phenotype is not observed 6 h postethanol exposure (Figure 1C).

**FIGURE 3:**
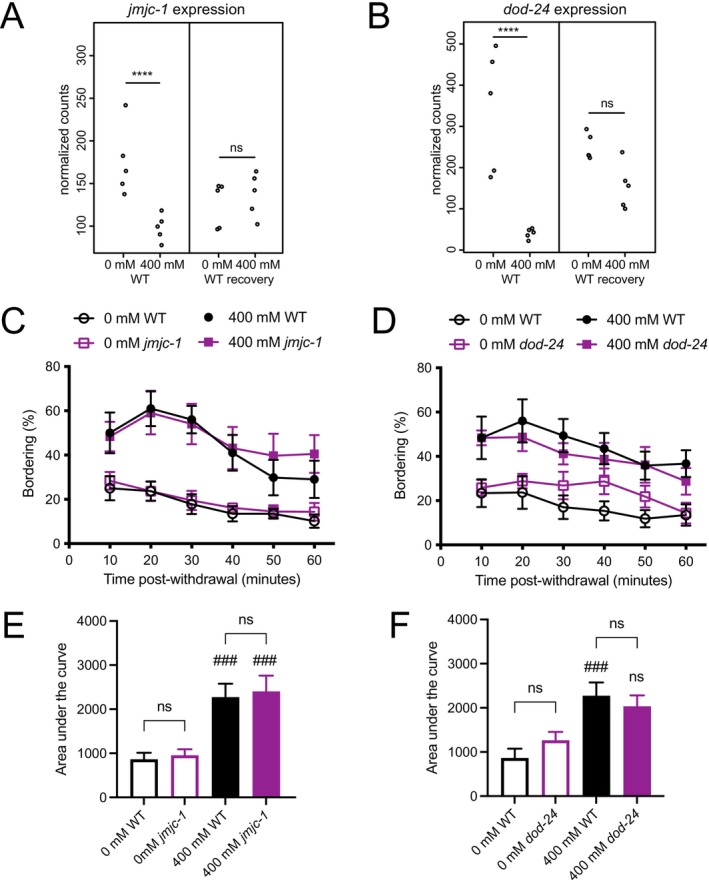
*npr‐1* pathway genes that are regulated following prolonged ethanol exposure. (A, B) Normalized expression of *jmjc‐1* (A) and *dod‐24* (B) in wild‐type RNA‐sequencing samples harvested immediately postexposure (0 h post‐exp) or 6 h postexposure (6 h post‐exp); both genes have significantly reduced expression immediately following ethanol exposure that is not maintained 6 h postexposure. Adjusted *p*‐value from DESeq2 analysis; ns, not significant; ****p ≤ 0.0001. (C, D) Bordering assays were performed on wild type (WT) in parallel with *jmjc‐1(tm3525)* (C) or *dod‐24(ok2629)* (D) mutants; wild type (black circles), mutant (purple squares) (*n* = 6). Filled symbols indicate prior exposure to 400 mM ethanol; open symbols indicate prior exposure to 0 mM ethanol. (C, D) Wild*‐*type, *jmjc‐1(tm3525)*, and *dod‐24(ok2629)* ethanol‐treated worms demonstrated more bordering than untreated worms; this is WIB. Statistical comparisons were made using two‐way ANOVA; for the effect of treatment in (C): Wild type = 0.0081; *jmjc‐1* = 0.0086; for the effect of treatment in (D): Wild type = 0.0088; *dod‐24, p* = 0.0403. (E, F) Area under the curve (AUC) for assays in C, D. (E) Wild type and *jmjc‐1(tm3525)* had significant WIB (400 mM vs. 0 mM). (F) Wild type had significant WIB, while *dod‐24(ok2629)* did not. Statistical comparisons were made using one‐way ANOVA with Holm‐Šídák multiple comparisons post hoc tests; for the effect of treatment: ns, not significant; ^###^ *p* ≤ 0.001; for the effect of genotype: ns, not significant. Error bars indicate SEM.

We were particularly interested in gene expression changes that occur immediately following ethanol exposure, but that do not persist 6 h after removal from ethanol; these are likely to underlie WIB. This gene set includes 1870 genes, 897 of which are upregulated and 973 of which are downregulated in response to ethanol exposure. We performed GO term analysis using the biological process annotation set and identified overrepresented categories related to fatty acid metabolism, including “fatty acid beta‐oxidation” and “fatty acid biosynthetic process” (Figure 3H; File S3). Interestingly, the fatty acid desaturase gene, *fat‐1*, that we previously identified as an important regulator of the acute alcohol response (Raabe et al., 2014), was transiently upregulated following prolonged ethanol exposure. In addition, among the overrepresented GO terms was the “innate immune response,” which we have identified as a class of genes that is regulated by SWI/SNF chromatin remodeling complexes in adult neurons (Mathies et al., 2020).

### Neuropeptide Y signaling

Prolonged ethanol exposure produces an increase in bordering that phenocopies loss‐of‐function alleles of the neuropeptide Y‐like receptor encoded by *npr‐1* (Davies et al., 2004). A working model for the relationship between *npr‐1* and WIB is that ethanol activates the *npr‐1* pathway, leading to a compensatory downregulation of the pathway during prolonged ethanol exposure that produces increased bordering upon removal from ethanol. The mechanism by which *npr‐1* pathway activity is downregulated is not known. One simple possibility is that *npr‐1* pathway members are transcriptionally regulated during prolonged ethanol exposure, but *npr‐1* itself was not transcriptionally regulated in this paradigm (Davies et al., 2004). Using our new RNA‐sequencing dataset, we found no changes in the expression of *npr‐1* or in any of four core components of the *npr‐1* signaling pathway, *gcy‐35, gcy‐36, tax‐2, tax‐4*, following an 18‐h exposure to ethanol, although it is important to note that the statistical power to detect changes in expression of these five genes is very low due to their low level of expression (File S4). We expanded our analysis to include any genes that have described genetic, regulatory, or physical interactions with *npr‐1*. This resulted in a list of 44 genes in an extended *npr‐1* network (File S4). Two of these genes, *dod‐24* and *jmjc‐1*, had expression patterns that were suggestive of their involvement in WIB; both had reduced expression immediately off ethanol that was not maintained 6 h postexposure (Figure 3A,B). *dod‐24* and *jmjc‐1* are regulated in an *npr‐1‐*dependent manner in sensory neurons in response to pathogen exposure (Styer et al., 2008); we therefore investigated them as good candidates to be mediators of the *npr‐1* effect on WIB.

If the extended ethanol‐induced decrease in *dod‐24* or *jmjc‐1* expression is important in WIB, then loss of function of these genes should decrease ethanol‐induced bordering, and the mutations may phenocopy WIB in the absence of ethanol exposure. To test this idea, we examined *dod‐24* and *jmjc‐1* mutants using the bordering assay. We used deletion mutations that are likely to result in a strong loss of function: *jmjc‐1(tm3525)* removes 429 bp resulting in a severely truncated JMJC*‐*1A protein; it has no effect on JMJC*‐*1B and *dod‐24(ok2629)* removes 401 bp of exon 2, truncating the protein after amino acid 97. Like wild type, both *jmjc‐1(tm3525)* and *dod‐24(ok2629)* showed an increase in bordering following ethanol exposure (Figure 3C,D). To compare the amount of bordering in the different strains and conditions, we used area under the curve (AUC) as a measure of cumulative bordering (Figure 3E,F). *jmjc‐1(tm3525)* mutants had comparable levels of bordering to wild type, both in the presence and absence of ethanol (Figure 3E), suggesting that *jmjc‐1* is not mediating WIB. In contrast, *dod‐24(ok2629)* mutants displayed qualitatively more bordering in the absence of ethanol and less bordering upon ethanol withdrawal than wild type (Figure 3F), and *dod‐24* mutants did not show statistically significant WIB when assessed using AUC.

We tested the possibility that *dod‐24* is required for bordering behavior itself, rather than for WIB in particular. *npr‐1* mutants show a strong bordering phenotype (de Bono & Bargmann, 1998). We generated a *dod‐24; npr‐1* double mutant and assessed its bordering behavior and found that *dod‐24* does not suppress the bordering behavior of *npr‐1* mutants in the absence of ethanol, demonstrating that the function of *dod‐24* is not essential for all bordering behavior (Figure S2A). Another possibility is that *dod‐24* may affect the accumulation or clearance of ethanol, and therefore affect WIB through changing the severity of the withdrawal from ethanol, so we examined tissue ethanol accumulation in *dod‐24* mutants. We found that *dod‐24* does not affect ethanol pharmacokinetics (Figure S3). Together, these results suggest that *dod‐24* contributes to WIB, but that it is not the sole gene mediating this behavioral change.

### SWI/SNF chromatin remodeling regulates withdrawal‐induced bordering

We interpret the WIB phenotype as revealing physiological changes that occurred during the prolonged ethanol exposure. There is growing evidence that epigenetic regulation underlies some of the physiological changes that occur following chronic alcohol use (reviewed in Berkel & Pandey, 2017; Cruise et al., 2023). We have previously found that SWI/SNF chromatin remodeling complexes regulate behavioral responses to acute alcohol exposure in *C. elegans* independent of affecting ethanol pharmacokinetics (Mathies et al., 2015), so we reasoned that they might also be mediating behavioral responses to prolonged ethanol exposure.

SWI/SNF complexes can be classified as BAF or PBAF, based on their subunit composition (Kwon & Wagner, 2007; Mohrmann et al., 2004; Mohrmann & Verrijzer, 2005). We previously defined the functions of all SWI/SNF subunits in the acute alcohol response in *C. elegans* and found that three PBAF subunits, SWSN*‐*7, SWSN*‐*9, and PHF*‐*10, were required for the development of acute functional tolerance (AFT) to ethanol (Mathies et al., 2015). Because PBAF is required for acute tolerance to ethanol, we hypothesized that it may also be important for the response to prolonged ethanol exposure. To examine the function of PBAF in WIB, we used *swsn‐9* as a representative member of the complex because there is a homozygous viable, strong loss‐of‐function deletion allele of the gene, *ok1354* (Large & Mathies, 2014; Mathies et al., 2015).

**FIGURE 4:**
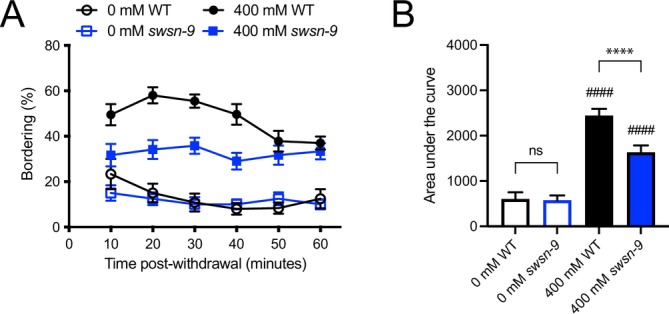
SWI/SNF PBAF subunit SWSN*‐*9 is required for normal levels of WIB. (A) Bordering assays were performed with wild‐type N2 (WT, black circles) and *swsn‐9(ok1354)* (blue squares). Filled symbols indicate prior exposure to 400 mM ethanol; open symbols indicate prior exposure to 0 mM ethanol. (B) Both wild‐type and *swsn‐9(ok1354)* worms bordered significantly more following ethanol exposure when compared with untreated worms; this is WIB. Statistical comparisons were made using mixed‐effects analysis; for the effect of treatment: Wild type, *p* ≤ 0.0001; *swsn‐9, p* = 0.0008. (B) Area under the curve for assay in B. Both wild type and *swsn‐9(ok1354)* had significant WIB (400 mM vs. 0 mM). Wild*‐*type and *swsn‐9(ok1354)* worms had similar levels of bordering following 0 mM exposure, but withdrawal‐induced bordering was significantly lower in *swsn‐9(ok1354)* than in wild type. Error bars indicate SEM. Statistical comparisons were made using one‐way ANOVA; for the effect of treatment: ^####^ *p* < 0.0001; for the effect of genotype: ns, not significant; ****p ≤ 0.0001.

We performed bordering assays on *swsn‐9* mutants in parallel with wild‐type N2 (Figure 4A). Both N2 and *swsn‐9(ok1354)* worms showed an increase in bordering behavior following removal from ethanol. *swsn‐9(ok1354)* appeared to border less than wild type upon withdrawal from 400 mM ethanol. To examine this possibility, we used AUC to compare bordering in each of the genotypes and conditions (Figure 4B). N2 and *swsn‐9* mutants had a similar amount of bordering in the absence of ethanol and both genotypes bordered significantly more upon withdrawal from ethanol. However, *swsn‐9* mutants bordered significantly less than wild type following withdrawal from 400 mM ethanol, indicating that *swsn‐9* is required for normal levels of WIB.

We found that loss of *swsn‐9* does not affect ethanol pharmacokinetics (Figure S3), and it does not suppress *npr‐1‐*mediated bordering (Figure S2B), indicating that the effect of *swsn‐9* on WIB is specific to the bordering induced by withdrawal from extended ethanol exposure.

### *swsn‐9* regulates genes that may be important for WIB

SWI/SNF complexes regulate gene expression by altering chromatin structure, so we reasoned that *swsn‐9* may be regulating genes that are important for the physiological changes that underlie WIB. To identify these genes, we examined gene expression in *swsn‐9(ok1354)* mutants following withdrawal from either 0 mM or 400 mM ethanol. We performed five biological replicates on different days and 0 mM and 400 mM ethanol exposures were done in parallel. The worms were harvested immediately after ethanol exposure and RNA‐sequencing libraries were prepared and sequenced by GeneWiz (South Plainfield, NJ).

**FIGURE 5:**
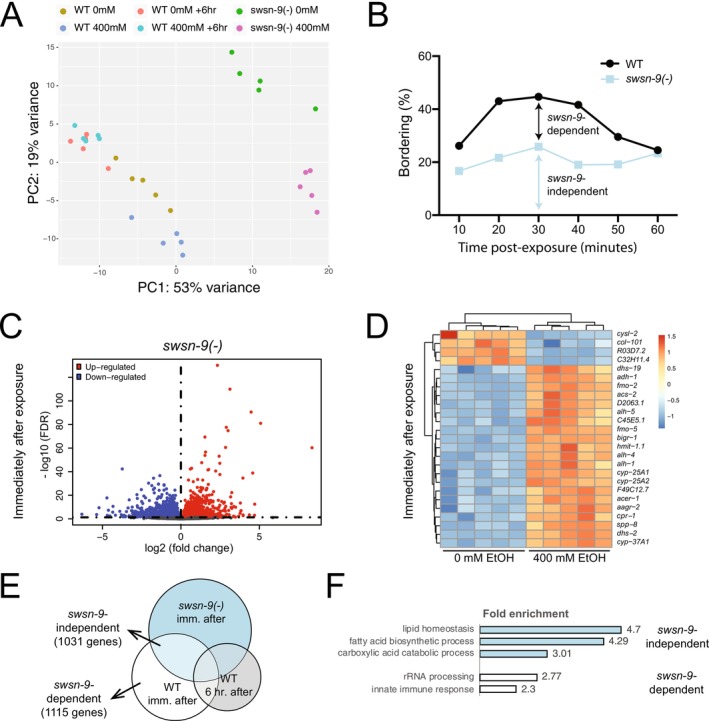
*swsn‐9* is required for some ethanol‐induced gene expression changes. Wild‐type or *swsn‐9* mutant animals were exposed to 0 or 400 mM ethanol for 18 h. RNA sequencing was performed immediately following exposure, or after a 6‐h withdrawal period. (A) Principal component analysis with gene expression plotted relative to the first two principal components (PC1 and PC2); replicates group together. Wild‐type N2 (WT) and *swsn‐9* mutants are separated by PC1, while control (0 mM ethanol) and treatment (400 mM ethanol) are distinguished by PC2. The difference between samples treated with 0 mM versus 400 mM ethanol is more pronounced for *swsn‐9* mutants than it is for wild‐type worms. (B) We expect gene expression differences that are responsible for WIB to fall into two classes: *swsn‐9*‐independent changes explain WIB in *swsn‐9* mutants, and *swsn‐9‐*dependent changes explain the difference in WIB between wild‐type and *swsn‐9* mutants. (C, D) Gene expression results for *swsn‐9* mutant worms exposed to 400 mM ethanol compared with 0 mM ethanol. (C) Volcano plot depicting genes that are upregulated (red) or downregulated (blue). (D) Heat map of the top 25 differentially expressed genes with blue indicating lower and red indicating higher expression. (E) Venn diagram showing the overlap between ethanol‐induced DEGs in *swsn‐9* mutants immediately following ethanol exposure (imm. after) versus wild type, immediately following ethanol exposure (imm. after) or 6 h postexposure (6 h after). (F) Gene Ontology (GO) biological process terms that are overrepresented among *swsn‐*9‐dependent or *swsn‐9‐*independent DEGs.

To examine the correlation among our samples and replicates, we performed principal component analysis. We included our wild*‐*type N2 gene expression data in this analysis so that comparisons could be made across all genotypes and treatments. We found that our replicates grouped together and that the difference between genotypes accounted for 53% of the variance (PC1), while treatment accounted for 19% of the variance (PC2) in the dataset (Figure 5A).

Our behavioral analysis suggests that genes underlying WIB fall into at least two classes: (1) genes that are mediating WIB in *swsn‐9* mutants (*swsn‐9‐*independent) and (2) genes that are responsible for the difference in the degree of WIB between wild‐type and *swsn‐9* mutants (*swsn‐9‐*dependent) (Figure 5B). We compared gene expression in *swsn‐9* mutants exposed to 400 mM or 0 mM ethanol and found 4274 genes that were differentially expressed between treatment and control (FDR < 0.05; fold change >1); more genes were downregulated (2605) than were upregulated (1669) in response to ethanol exposure (Figure 5C). When we re‐analyzed our wild*‐*type samples with the *swsn‐9* mutant samples, we identified 2645 DEGs immediately off ethanol and 1460 DEGs 6 h postexposure. Importantly, the DEGs identified in this analysis are largely overlapping with our previous analysis. All DEG lists are in File S5. The top 25 ethanol‐responsive genes are shown in the heatmap (Figure 5D); most of the genes are upregulated following ethanol exposure. We characterized the overlap between ethanol‐induced DEGs in wild‐type and *swsn‐9* mutants (Figure 5E). We expect to find *swsn‐9‐*dependent gene expression changes among the 1031 genes that are unique to N2 and *swsn‐9‐*independent gene expression changes among the 1078 genes that are common to wild‐type and *swsn‐9* mutants and that change in the same direction in both genotypes. All DEG lists are in File S5. In both cases, we excluded genes whose expression remains changed 6 h postexposure to ethanol because we do not observe WIB at this time. We performed GO term analysis on the *swsn‐9‐*dependent and *swsn‐9‐*independent DEGs and found that the *swsn‐9‐*dependent genes have an overrepresentation of genes in the GO “innate immune response” category and *swsn‐9‐*independent genes have an overrepresentation of genes in the GO “fatty acid biosynthetic process” category (Figure 5F, File S6).

## DISCUSSION

Prolonged exposure to alcohol causes profound changes in gene expression in the human brain, and such persistent gene expression changes are thought to be important in the development of alcohol dependence and in the etiology of AUD (e.g., Willis et al., 2025). We sought to identify gene expression changes that underlie the development of physiological dependence on ethanol in *C. elegans*. Following an extended (18 h) exposure to ethanol, worms develop physical dependence on ethanol. We observe this dependence by changes in behavior when the ethanol is withdrawn; we find that ethanol‐withdrawn worms transiently increase their preference for being located in the thicker border of a bacterial lawn, a behavior we call “withdrawal‐induced bordering” (WIB) (Davies et al., 2004). Here, we investigated the mechanisms underlying WIB, concentrating on gene expression changes following prolonged ethanol exposure, to identify genes mediating the behavioral effects of ethanol withdrawal. We focused on differential gene expression observed after 18 h of ethanol exposure, when WIB is apparent, but that is lost by 6 h of withdrawal, when the WIB behavior was no longer detectable. We performed RNA sequencing on animals immediately following 18 h of ethanol exposure, and following 18 h of ethanol exposure and 6 h of withdrawal. Two hundred and ninety‐one genes were differentially expressed persistently upon removal from ethanol and after 6 h in the absence of ethanol; we hypothesize that some of these gene expression changes may underlie other behavioral consequences of prolonged ethanol exposure that were not measured here. We found 1870 genes whose expression changed following an 18‐h ethanol exposure and was normalized after a 6‐h recovery period in the absence of ethanol.

### SWI/SNF‐dependent and independent gene expression changes identify different biological processes

The SWI/SNF complex gene *swsn‐9* is required for normal levels of WIB and the full gene expression response to prolonged ethanol exposure. About half of the genes that are normally differentially expressed following ethanol exposure are dependent on *swsn‐9* for their regulation and about half of the bordering that is induced by withdrawal from prolonged ethanol exposure is dependent upon *swsn‐9*. This suggests that *swsn‐9*, and by extension the PBAF complex, regulates genes that explain approximately half of the ethanol withdrawal phenotype. Interestingly, GO analysis identifies innate immune genes as a functional group that is overrepresented among the *swsn‐9‐*dependent gene expression changes. We have previously demonstrated that innate immune genes are overrepresented among genes that are regulated by SWI/SNF complexes in adults and in neurons (in the absence of ethanol) (Mathies et al., 2020). To ask whether the same genes might be mediating the SWI/SNF effect on acute and chronic alcohol responses, we compared the innate immunity genes identified in the two studies. We found two genes in common: F01D5.1 and F01D5.5 are negatively regulated by SWI/SNF in adult neurons (Mathies et al., 2020) and had reduced expression following prolonged ethanol exposure that was dependent on *swsn‐9* (this study). Increased F01D5.1 and F01D5.5 expression is associated with reduced AFT in SWI/SNF mutants and reduced expression is associated with *swsn‐9‐*dependent WIB following prolonged ethanol exposure. These findings raise the possibility that F01D5.1 and F01D5.5 may be important for the behavioral effects of acute and prolonged (chronic) alcohol exposure.

### Regulation of *npr‐1/NPY* pathway components

Bordering behavior has been extensively characterized in *C. elegans* (reviewed in Portman, 2020). In the wild, one mechanism by which *C. elegans* localize themselves beneath the surface of the soil is through a preference for the lower oxygen environment underground. In the laboratory, the border of a bacterial lawn is thicker and has a lower local oxygen concentration; this is the cue that promotes bordering behavior. The *npr‐1* NPY receptor‐like signaling pathway is a key player in modulating the amount of bordering behavior demonstrated by animals. The increased bordering seen following withdrawal from ethanol phenocopies the effect of loss‐of‐function mutations in *npr‐1* (Davies et al., 2004). Acute exposure to ethanol suppresses bordering behavior, suggesting that ethanol potentiates the activity of the *npr‐1* signaling pathway (Davies et al., 2004). Together, these observations led us to a model in which prolonged ethanol exposure‐induced potentiation of *npr‐1* signaling causes a compensatory downregulation of the *npr‐1* signaling pathway. This downregulation is revealed as WIB when animals are withdrawn from extended ethanol. We examined the expression of known *npr‐1* signal transduction pathway components and genes that are differentially regulated in an *npr‐1*‐dependent manner and found two genes, *jmjc‐1* and *dod‐24*, whose expression was decreased by prolonged ethanol exposure but had returned to normal after 6 h of withdrawal. If reduced expression of either gene is responsible for the increased bordering upon withdrawal, we would expect loss‐of‐function mutants to have less or no increase in bordering following ethanol withdrawal because gene function cannot be reduced further. We also may observe increased bordering in the absence of ethanol because loss of gene function may mimic the ethanol‐induced inhibition of gene expression. We found that a mutation in *jmjc‐1* did not alter the behavior of animals in the WIB assay, suggesting that *jmjc‐1* is not involved in this withdrawal phenotype. It should be noted, however, that the *jmjc‐1* mutant only affects JMJC*‐*1A, so it remains possible that JMJC*‐*1B may play a role in WIB.

### *dod‐24* is required for WIB

We identified a role for the gene encoding the CUB‐like domain‐containing protein DOD‐24 in ethanol‐induced withdrawal behavior. The increase in bordering after withdrawal from extended ethanol exposure appears to be attenuated in *dod‐24* mutants; WIB was statistically significant by ANOVA (effect of treatment), but not by area under the curve comparisons. This lack of significance for the area under the curve measure is partially driven by a trend toward elevated basal bordering behavior shown by the untreated animals. Both of these *dod‐24* phenotypes of increased basal bordering and reduced WIB are consistent with a gene whose expression is reduced following ethanol exposure and that underlies WIB, suggesting that *dod‐24* has a role in WIB. While the molecular function of DOD*‐*24 has not yet been described, several studies suggest a role for DOD*‐*24 in neurons. Most recently, Weng et al. (2024) demonstrated that *dod‐24* is important for maintaining proper neuronal function in aged animals and suggested that this role is neuron‐autonomous. Two additional studies point to a possible function for *dod‐24*: Mack et al. (2022) found that *dod‐24* and several Δ9 fatty acid desaturases are regulated by the minibrain‐related kinase *mbk‐1*. *mbk‐1* mutants are defective in the ability to withstand heat shock. Okahata et al. (2024) directly tested *dod‐24* for a role in temperature tolerance using a cold shock paradigm; *dod‐24* mutant animals are more sensitive to cold than wild type, further demonstrating that *dod‐24* has a function in the ability to respond to temperature changes. One mechanism by which animals modulate their responses to temperature is through regulation of the structure of their lipid membranes; this can be achieved through modulation of the fatty acid composition of the membranes (e.g., Devkota et al., 2021). We have previously demonstrated that both temperature and genes that are involved in lipid metabolism can modulate acute ethanol responses (Bettinger et al., 2012; Mathies et al., 2015; Raabe et al., 2014). Furthermore, the development of acute functional tolerance to ethanol requires eicosapentaenoic acid, which is generated by the function of the *fat‐1* gene (Raabe et al., 2014), and here, we observed that *fat‐1* is regulated in response to extended ethanol exposure. Together, these observations may suggest that one part of the development of physical dependence on ethanol is the manipulation of membrane structure and function through changes in fatty acid composition. We hypothesize that *dod‐24* may influence neuronal membrane function to affect the WIB phenotype. Importantly, loss of *dod‐24* did not eliminate WIB, indicating that there are additional, as yet unidentified mechanisms underlying WIB.

## FUNDING INFORMATION

This research was supported by grants from the NIH to LDM and JCB (R01 AA024482) and to JCB and AGD (P50 AA022537). Some strains were obtained from the *Caenorhabditis* Genetics Center, which is funded by the NIH Office of Research Infrastructure Programs (P40 OD010440).

## CONFLICT OF INTEREST STATEMENT

The author declares no conflicts of interest.

## Supporting information


Figure S1

Figure S2

Figure S3

File S1

File S2

File S3

File S4

File S5

File S6

Video S1

## Data Availability

Strains are available upon request. The RNA‐sequencing dataset generated during this study is available in the NCBI Sequence Read Archive (SRA), accession number PRJNA1263870, and raw counts are available in the NCBI Gene Expression Omnibus (GEO), accession number: GSE297802.
