# Supplementary material for: SWI/SNF complexes modulate gene expression and the development of physical dependence to ethanol
Source: Alcohol Clin Exp Res (Hoboken). 2026 Jan 12;50(1):e70223. doi: 10.1111/acer.70223 (PMC12796780; doi:10.1111/acer.70223)
Supplement: Supplementary file 1 — Figure S1 [file ACER-50-0-s005.pdf]

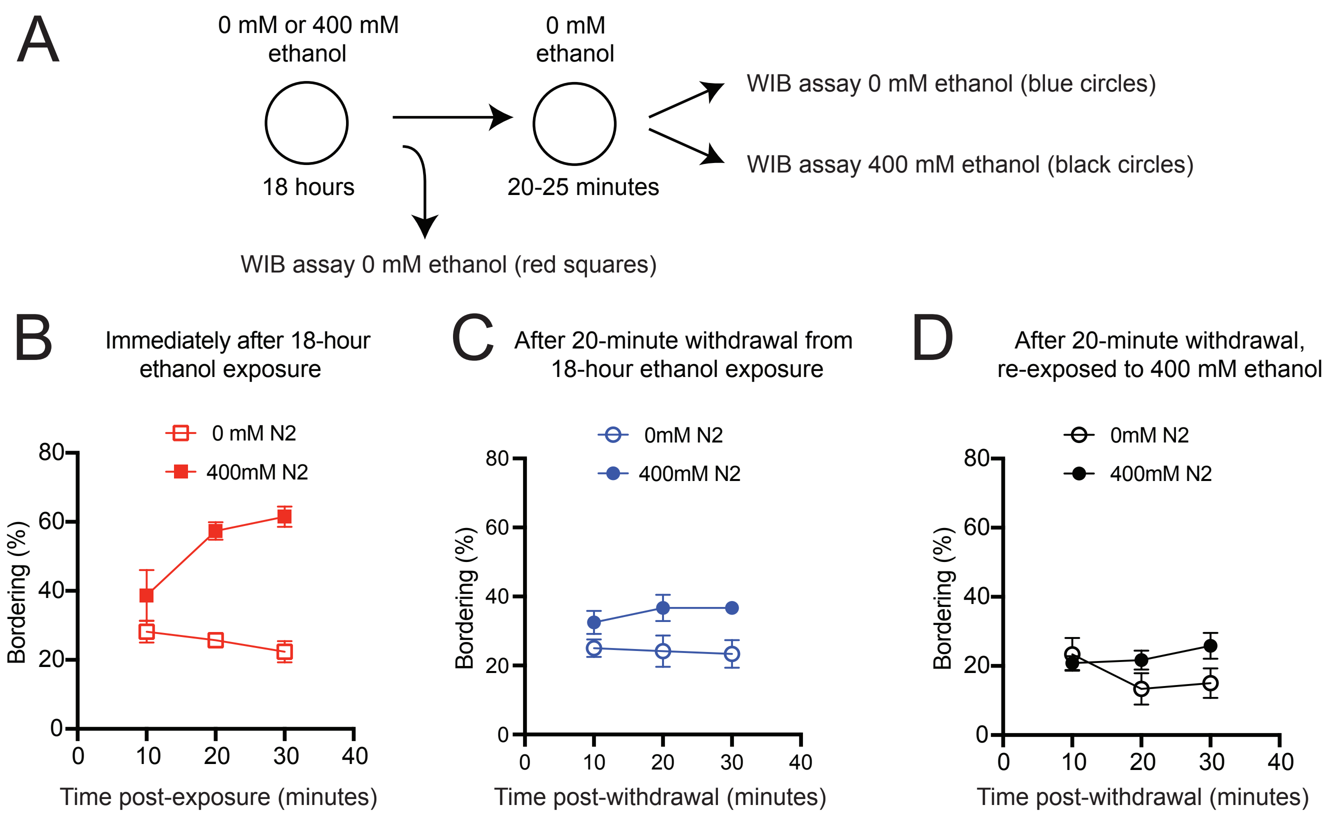

Figure S1. Re-exposure to ethanol suppresses WIB.

A. Experimental design. Wild-type N2 worms were exposed to 0 mM or 400 mM ethanol for 18 hours. Twenty worms were assayed immediately after exposure (B), while the remaining worms underwent a 20-25 minute withdrawal period before being assayed on plates containing either 0 mM ethanol (continued withdrawal) or 400 mM ethanol (re-exposure). B-D.Bordering assays were performed immediately after removal from ethanol (A) or following a 20-25 minute withdrawal period (B-C). Filled symbols indicate prior 18 hour exposure to 400 mM ethanol; open symbols indicate prior exposure to 0 mM ethanol. B. Worms that were assayed immediately after exposure had significant WIB ($p \leq 0.0001$). C-D. Worms that experienced withdrawal and were assayed on plates with 0 mM ethanol (continued withdrawal) showed a significant effect of treatment ($p = 0.028$), whereas those re-exposed to ethanol did not. Error bars indicate S.E.M. Statistical comparisons were made using two-way ANOVA.
