# Supplementary material for: SWI/SNF complexes modulate gene expression and the development of physical dependence to ethanol
Source: Alcohol Clin Exp Res (Hoboken). 2026 Jan 12;50(1):e70223. doi: 10.1111/acer.70223 (PMC12796780; doi:10.1111/acer.70223)
Supplement: Supplementary file 2 — Figure S2 [file ACER-50-0-s003.pdf]

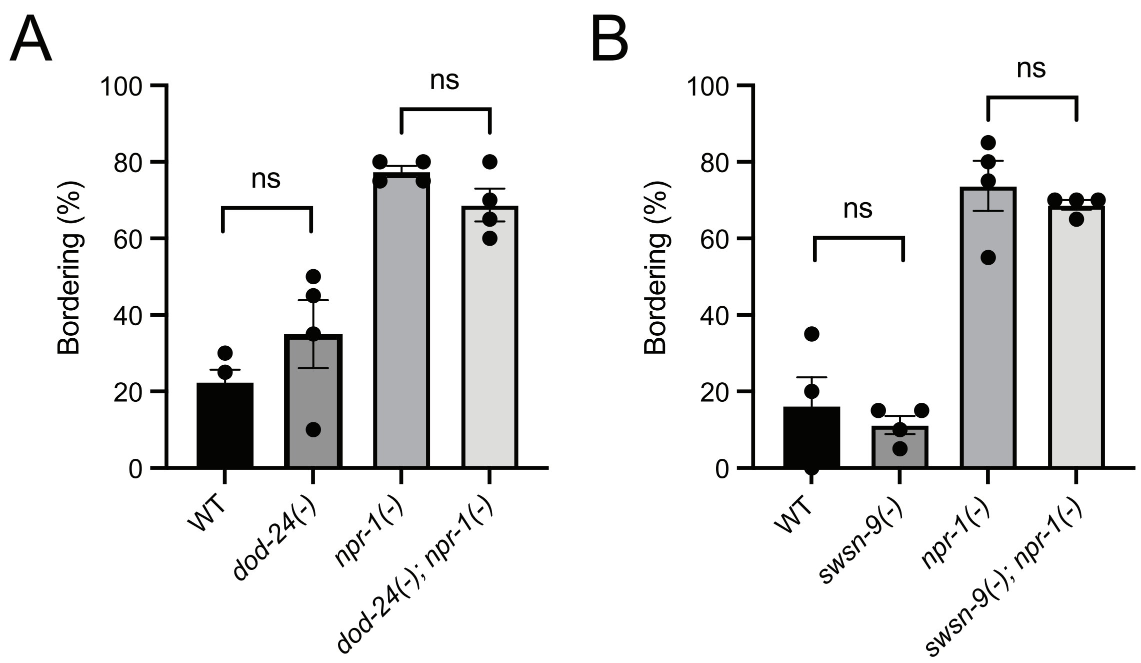

Figure S2. *dod-24* and *swsn-9* do not broadly affect bordering behavior.
The ability to suppress bordering in *npr-1(ky13)* mutants was assessed for *dod-24(ok2629)* (A) and *swsn-9(ok1354)* (B). Each mutant was tested alone and in combination with *npr-1* mutants. Wild-type N2 and *npr-1* single mutant worms were included for comparison. A. *dod-24* mutants showed a slight but not statistically significant increase in basal bordering behavior and did not suppress bordering in *npr-1* mutants. B. *swsn-9* mutants displayed wild-type levels of bordering behavior and were similarly not able to suppress bordering in *npr-1* mutants. Statistical comparisons were made using paired Student's t-tests; ns, not significant.
