# Supplementary material for: SWI/SNF complexes modulate gene expression and the development of physical dependence to ethanol
Source: Alcohol Clin Exp Res (Hoboken). 2026 Jan 12;50(1):e70223. doi: 10.1111/acer.70223 (PMC12796780; doi:10.1111/acer.70223)
Supplement: Supplementary file 3 — Figure S3 [file ACER-50-0-s004.pdf]

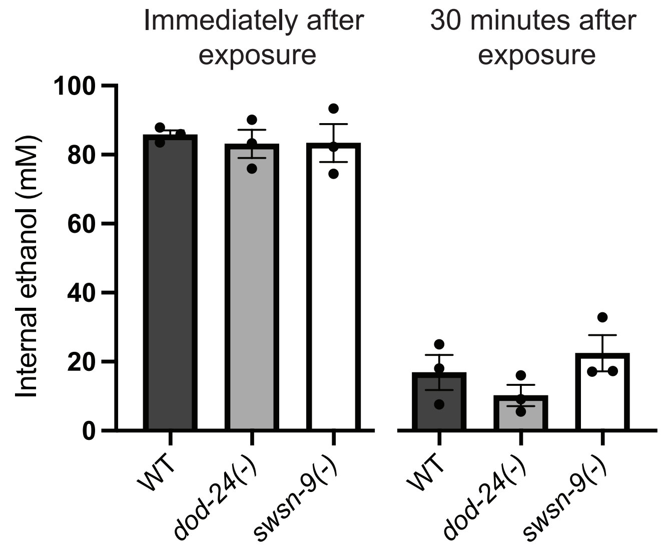


**Figure S3. *dod-24* and *swsn-9* do not affect tissue ethanol concentrations.**
Tissue ethanol concentrations were measured for wild-type N2, *dod-24(ok2629)*, and *swsn-9(ok1354)* mutants following 18-hour exposure to 400 mM ethanol and after a 30-minute withdrawal period. 100 worms were collected immediately after exposure, and an additional 100 worms was transferred to plates containing 0 mM ethanol for 30 minutes prior to collection. Ethanol concentration was measured for all samples, and internal tissue concentrations were calculated. Statistical comparisons were made using two-way ANOVA; no significant effect of genotype was detected.
