# Supplementary material for: SWI/SNF complexes modulate gene expression and the development of physical dependence to ethanol
Source: Alcohol Clin Exp Res (Hoboken). 2026 Jan 12;50(1):e70223. doi: 10.1111/acer.70223 (PMC12796780; doi:10.1111/acer.70223)
Supplement: Supplementary file 10 — Video S1 [file ACER-50-0-s009.zip › Video_legend.docx]

**Video S1** Representative withdrawal-induced bordering assay. Worms were treated with 400 mM (left ring) or 0 mM (right ring) ethanol for 18 hours prior to the start of the assay.
